# On Cynanche Trachealis

**Published:** 1824-10

**Authors:** George Meyers


Art. VII.—*On Cynanche Trachealis.* By George ~~M[illegible]~~ Rogers, M.D.

Although Etmuller, Cullen, and other nosologists, have given correct definitions of the symptoms of this disease, it will be, perhaps, of more practical importance to be content with a more simple detail of the symptoms, as connected with the relative situation of the disease in question. The seat of this disease, then, I would say, is in the cavity of the trachea, or windpipe, and arises from high inflammatory action, whereby an extensive morbid secretion is thrown out from the mucous membrane lining the internal surface of that organ. This very soon becomes of a thick gelatinous nature, and consequently prevents the accustomed passage of air to the lungs; and, in proportion to its continuance or increase, entirely obliterates the canal, and the patient, of course, becomes suffocated.

The attack of this very formidable disease is particularly sudden, and comes on with a sense of uneasiness about the throat, constriction of the fauces, and a difficulty of respiration, which are commonly supposed to be the consequence of an ordinary sore-throat. This difficulty of breathing is attended with a peculiarly-marked hoarseness and croaking sound; and, upon examining the fauces, the velum pendulum palati, and the whole of the neighbouring parts, are observed to be parched and dry. The commencement of the attack is not accompanied with any completely-marked cold shivering fit, as is usually the case in most instances of phlogosis; or, if it be, the patient is too much oppressed with the foregoing symptoms to allow him to name that as the precursor of them. It generally happens that the patient has been labouring under the influence of this disease many hours before medical aid is had recourse to; and, if two or three days have elapsed before proper assistance is procured, the disease has, in all probability, so rapidly advanced, and so thoroughly formed itself, as to baffle the utmost exertions of the practitioner. At this period the countenance becomes ghastly, the lips pale or livid, the pulse depressed and tremulous, the extremities cold, and the ability to swallow even liquids impracticable.

When called upon under these most distressing and alarming circumstances, it is to be feared little can be done, for the disease has arrived at that pitch of despair that scarcely any human interposition will ever avail, even in rendering a palliative. We are nevertheless bound by the laws of humanity to attempt some means of relief; and the annexed cases will explain how far we are justified in having recourse to prompt and active measures, since, in one of them in particular, it must be allowed there did not appear to be the smallest prospect of success, and yet ultimately the patient recovered.

CASE I.—William Josselyn, ætatis eleven, had been attacked with sore-throat and hoarseness three days before I saw him, and had merely been taking a little purgative medicine, and using a common vinegar-gargle, by his mother's desire. I saw him on the fifth day of the attack, when he was labouring under urgent symptoms of cynanche trachealis, and to so alarming a degree, that I ventured to predict a fatal termination within twenty-four hours. Leeches and a blister to the throat were immediately had recourse to, and four grains of calomel were given every second hour; but, within ten hours from the time of my first visiting him, he breathed his last.

The following morning I obtained permission to inspect the part; and, upon laying open the trachea, from the cricoid cartilage to its termination in the bronchial tube, I found the whole cavity so completely blocked up with dense coagulable lymph, that I could with difficulty pass the handle of my knife along. I carefully at length separated a large portion of it, and was struck with the florid appearance of the internal coat of the trachea, much resembling scarlet cloth as to its colour. It will be observed, this disease had been of five days' standing only, and must indisputably prove the rapidity with which it advances, and the impracticableness of removing it when thus formed and fixed. This dissection likewise throws additional light upon the treatment necessary to be adopted and pursued; and, although it may not afford any reason for employing a fresh or novel plan, most unquestionably tells us that our efforts hitherto made must be more vigorously followed up.

CASE II.—John Banks, ætatis forty, had been attacked with decided marks of cynanche trachealis for upwards of forty-eight hours previous to my being called in. I found him with very difficult breathing, accompanied with a stridulous noise; the appearance of the fauces parched and dry; countenance ghastly; lips of a deep livid hue; pulse extremely laboured and low, not exceeding forty; extremities cold; bowels costive; tongue foul, and covered with a thick white incrustation; great difficulty in swallowing. Leeches were immediately applied in a line with the tracheal tube, and not transversely; immediately afterwards, a blistering-plaster in the same direction; four grains of the submuriate of quicksilver were taken, and desired to be repeated every fourth hour.

The following day, July 27th, except the bowels having been relieved, all the symptoms were aggravated. Two grains of the submuriate were now ordered every second hour. Fomentations, and afterwards friction to the feet and legs. Nourishment, tea and gruel only.

28th.—The difficulty of breathing somewhat relieved, and the same planto be punctually persevered in.

29th.—Much improved; the breathing being more natural, and free from the stridulous sound; the pulse more regular, about sixty; the countenance a little animated, and the capability of swallowing accomplished with tolerable facility. The submuriate was now ordered to be continued every fourth hour, with the fomentations and friction.

30th.—Much better, and sitting up in his chair; the salivary glands excited, and spitting up thick coagulated mucus. The submuriate, in doses of ten grains, was now ordered twice in the day only.

31st.—Apparently quite well. Left off his medicine, and kept the bowels open with laxative pills, merely as occasion required. Had no relapse.

Case III.—William Goddard, ætatis two, attacked with complete symptoms of cynanche trachealis about twelve hours before I was called in; and, although the general symptoms were not quite so distressing as in the preceding case, yet the croaking sound was more considerable and conspicuous. Leeches were immediately applied as in the last case, succeeded by a blister in a longitudinal direction. Two grains of the submuriate of quicksilver were ordered every second hour; and the child was to be immersed in a warm bath, a little above the hip, every fourth hour, and to be kept nourished entirely by tea and thin gruel.

The day following (January 29th), the breathing was no worse, and the same plan was ordered to be regularly continued.

30th.—Better; and a quantity of saliva produced, with thick phlegm frequently thrown up. The medicine to be continued every six hours only.

31st.—Much relieved from the laborious breathing, as well as from the croaking sound; the countenance more lively; able to swallow freely; bowels pretty well.

February 1st.—The child quite free from disease, sitting up; and ordered a little rhubarb powder, to be taken occasionally, if the state of the bowels should require it.

5th.—The child continues quite well.

Case IV.—Elizabeth Powell, ætatis nine weeks, seized with true marks of cynanche trachealis ten or twelve hours only previous to my seeing her. The breathing was laborious, and the croaking sound very considerable. Half a grain of the submuriate of quicksilver was ordered every third hour, and semicupium to be adopted every second, third, or fourth hour, according to the violence or increase of the symptoms.

The following day (April 28th), some little improvement in breathing, and an evident decrease of the croaking sound. The same plan to be continued as before.

29th.—The urgent symptoms all much relieved. The submuriate to be repeated every six hours only, and the semicupium to be employed night and morning.

30th.—The child now breathes with great facility, and is altogether much improved.

May 1st.—Quite well, and free from the disease altogether.

From the view I had taken of the morbid condition of the internal lining of the trachea in Case I., I am induced to believe that no relief can be obtained in this disease, unless it can be prevented in time from going on so far as to obstruct the passage of air to the lungs; or, unless, when this obstruction is already formed, it can be removed by artificial means, or by the power of medicine internally administered. The former can only be accomplished by the operation of tracheotomy, which, although perhaps it might afford trifling and temporary relief, would eventually fail as a remedy; but, as it has been attempted by the late Mr. Chevalier and others, it must be admitted that it is a subject worthy of some consideration. According to the latter, the adhibition of mercury appears to have produced wonderful effects in mitigating and relieving all the symptoms, very soon after it has been employed, for twenty-four hours in succession. Friction along the course of the tracheal tube with a liniment of the submuriate of quicksilver, would also be highly proper; but this surface is obliged to be covered with leeches and a blistering plaster immediately, to suppress the active inflammation going on, so that there is no opportunity of employing that process to advantage. It would appear that the mercury acts more immediately upon the parts connected with the seat of the disease, because a copious discharge of froth and saliva is produced, and frequently even thick flaky mucus is thrown off from the throat, after the second day of its repeated and continued adhibition; so that it would almost be presumed that the trachea itself is affected by it, and, in consequence, the morbid secretion there generated is loosened and hawked up, as in Cases II. and III.

By the view of Case I. post mortem, I am inclined to feel convinced of the necessity of applying friction, leeches, and blisters *longitudinally*, as in Cases III. and IV., and not circularly, as has generally been the plan hitherto adopted. Semicupium, or fomentations, with subsequent friction, every second, third, or fourth hour, according to the urgency of symptoms, are highly useful; and the diet should consist of little else than tea and gruel.

*Manningtree, Essex; August*, 1824.

