# Errata in Our Number for October

**Published:** 1824-12

**Authors:** 


Errata *in our Number for October.*
Page 298, for Dr. Meyers, read Dr. Rogers.

